# Formate hydrogenlyase in the hyperthermophilic archaeon, *Thermococcus litoralis*

**DOI:** 10.1186/1471-2180-8-88

**Published:** 2008-06-03

**Authors:** Mária Takács, András Tóth, Balázs Bogos, András Varga, Gábor Rákhely, Kornél L Kovács

**Affiliations:** 1 Department of Biotechnology, University of Szeged, Közép fasor 52. H-6726 Szeged, Hungary; 2 Institute of Biophysics, Biological Research Center, Hungarian Academy of Sciences, Szeged, Hungary; 3 Institute of Plant Biology, Biological Research Center, Hungarian Academy of Sciences, Szeged, Hungary

## Abstract

**Background:**

*Thermococcus litoralis *is a heterotrophic facultative sulfur dependent hyperthermophilic Archaeon, which was isolated from a shallow submarine thermal spring. It has been successfully used in a two-stage fermentation system, where various keratinaceous wastes of animal origin were converted to biohydrogen. In this system *T. litoralis *performed better than its close relative, *P. furiosus*. Therefore, new alternative enzymes involved in peptide and hydrogen metabolism were assumed in *T. litoralis*.

**Results:**

An about 10.5 kb long genomic region was isolated and sequenced from *Thermococcus litoralis. In silico *analysis revealed that the region contained a putative operon consisting of eight genes: the *fdhAB *genes coding for a formate dehydrogenase and the *mhyCDEFGH *genes encoding a [NiFe] hydrogenase belonging to the group of the H_2_-evolving, energy-conserving, membrane-bound hydrogenases. Reverse transcription linked quantitative Real-Time PCR and Western blotting experiments showed that the expression of the *fdh-mhy *operon was up-regulated during fermentative growth on peptides and down-regulated in cells cultivated in the presence of sulfur. Immunoblotting and protein separation experiments performed on cell fractions indicated that the formate dehydrogenase part of the complex is associated to the membrane-bound [NiFe] hydrogenase.

**Conclusion:**

The formate dehydrogenase together with the membrane-bound [NiFe] hydrogenase formed a formate hydrogenlyase (formate dehydrogenase coupled hydrogenase, FDH-MHY) complex. The expression data suggested that its physiological role is linked to the removal of formate likely generated during anaerobic peptide fermentation.

## Background

Hyperthermophilic microorganisms, growing optimally at or above 80°C, have been isolated from a variety of geothermally heated environments [1]. Almost all of them belong to the Archaea (Archaebacteria) domain [2]. The majority of them are strict anaerobs and obligately dependent upon elemental sulfur (S°), which serves as terminal electron acceptor leading to H_2_S production. In some of the heterotrophic species, capable to grow facultatively without S°, the fermentative utilization of sugars and peptides results in the formation of molecular hydrogen [3].

Hydrogenases responsible for hydrogen consumption and/or production belong to a diverse family of enzymes [4]. Numerous hydrogenases belonging to various classes of [NiFe] hydrogenases have been identified and characterized in the group of heterotrophic hyperthermophilic Archaea [4]. The [NiFe] hydrogenases are linked to wide variety of metabolic pathways in various microorganisms. Many of them catalyze hydrogen uptake but numerous [NiFe] hydrogenases produce hydrogen, as well. Their proposed physiological role is to maintain the pH and the redox balance of the cells [4,5]. The hydrogen evolving [NiFe] hydrogenases are multisubunit membrane-bound enzymes utilizing various electron donors like ferredoxins or polyferredoxins [6]. The prototype of the multimeric membrane-bound hydrogenases is the hydrogenase 3 in *Escherichia coli*, which is part of the formate hydrogenlyase complex coupling formate oxidation to proton reduction [7,8].

In this study we focused on the H_2 _metabolism of *Thermococcus litoralis*, which was isolated from a shallow submarine thermal spring [9]. Both *T. litoralis *and its close relative, *Pyrococcus furiosus *are well-known heterotrophic facultative sulfur dependent hyperthermophiles, members of the *Thermococcus *genus of the euryarchaeal order Thermococcales [10]. These microbes preferentially utilize sugars and/or peptides for growth: the glycolysis occurs via a modified Embden-Meyerhof pathway [11], while amino acids derived from peptides are metabolized by transaminases and four distinct 2-keto acid oxidoreductases into their corresponding coenzyme A derivatives [12]. Two acetyl-CoA synthetases transform the CoA derivatives to organic acids with concomitant substrate-level phosphorylation to form ATP [13]. Depending on the redox status of the cell, 2-keto acids can be decarboxylated to aldehydes which are further oxidized to carboxylic acids by aldehyde:ferredoxin oxidoreductase (AOR) [14,15]. Another enzyme, formaldehyde:ferredoxin oxidoreductase (FOR) is also thought to be involved in the catabolism of amino acids [15,16].

Two soluble heterotetrameric NAD(P)-reducing (Hyh1, Hyh2) and one energy conserving multisubunit membrane-bound (Mbh) [NiFe] hydrogenases were identified in *P. furiosus *[17-19]. Based on bioenergetics considerations the membrane-bound hydrogenase was suggested to be mostly responsible for the hydrogen evolving capacity of the cells while the soluble bidirectional hydrogenases were proposed to have redox fine tuning role [20,21].

So far, only a cytoplasmic [NiFe] hydrogenase (Hyh1) was characterized from *T. litoralis *[22], and evidences were found for the presence of the genes of the second soluble (Hyh2) (GenBank accession no.: EU024408) as well as the membrane-bound hydrogenase (Mbh) (our unpublished results). *T. litoralis *has been successfully used in a two-step fermentation system, where various keratinaceous wastes of animal origin were converted to biohydrogen [23]. In this system *T. litoralis *performed better than *P. furiosus*. Therefore, new alternative enzymes involved in peptide and hydrogen metabolism were hypothesized in *T. litoralis*.

Here, we report the isolation and characterization of an operon from *T. litoralis*, which codes for a complex that is composed of subunits which show high sequence similarity to the components of the non-energy-conserving formate hydrogenlyase (formate dehydrogenase coupled hydrogenase complex, FDH-MHY) system of *E. coli *[7]. Experimental evidences were provided that the formate dehydrogenase subunits were associated to the hydrogenase bound to the membrane, therefore it is suggested that they formed a formate hydrogenlyase complex. Usually, formate hydrogenlyases play a role in sugar metabolism however, the FDH-MHY complex in *T. litoralis *seems to be linked to the peptide metabolism.

## Results

### Isolation and characterization of the *fdh-mhy *operon

**Figure 1:**
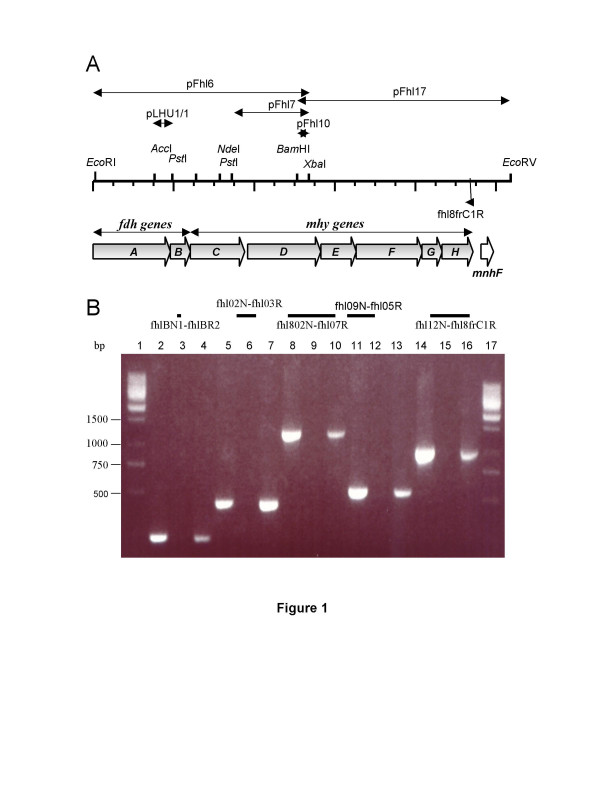
**The genomic organization and transcription of the *fdh-mhy *operon**. **A**. Restriction map of the isolated 10.5 kb long *T. litoralis *genomic fragment and the genetic organization of the *fdh-mhy *genes. The main subclones are indicated by arrows. **B**. The products of the RT-PCR experiments performed to prove that the *fdh-mhy *genes are located on a single mRNA. cDNA was made using the fhl8frC1R primer, and the amplified regions is indicated with bold lines. Lane 1,17: GeneRuler™ 1 kb DNA Ladder (Fermentas). Templates: lane 2,5,8,11,14: genomic DNA template, lane 3,6,9,12,15: RT(-) experiment lacking reverse transcriptase, lane 4,7,10,13,16: RT(+) experiment. Primers: lane 2–4: fhlBR2-fhlBN1, lane 5–7: fhl02N-fhl03R, lane 8–10: fhl802N-fhl07R, lane 11–13: fhl05R-fhl09N, lane 14–16: fhl8frC1R-fhl12N.

Two genes coding for proteins similar to the α and β subunits of various microbial formate dehydrogenases were identified during the isolation of *hyh-1 *operon of *T. litoralis *[22]. As formate dehydrogenases often form complexes with hydrogenases (formate dehydrogenase coupled hydrogenase, FDH-MHY), the genomic region downstream from these genes was investigated in detail (Table 1). A 10.5 kb genomic region was isolated in two steps from *T. litoralis *partial genomic DNA libraries (see Materials and Methods,) (Fig. 1A). Nine open reading frames could be identified; all of them were preceded by conserved ribosomal binding sites.

**Table 1:** Nucleotide sequence of the primers used in the RT-PCR experiments.

Primer	sequence
fhlBN1	5' CTCATCGAGACATCCGAACT 3'
fhlBR2	5' TGACTGCAGAGGTCGCACTT 3'
fhl02N	5' ACTGCTGCTGAGCCTGATTC 3'
fhl03R	5' TCAGTGGTGTAAATAGAGAC 3'
fhl05R	5' CCAGTCGTCAGGAAGTATCA 3'
fhl07R	5' TATCGCGTCCGATCCGTAGG 3'
fhl802N	5' TTGTTCAGAAGGACATAA 3'
fhl805R	5' CCTTTATGATGCCGTCAA 3'
fhl806N	5' GAGCCTGATTCTGGTGGA 3'
fhl8frC1R	5' CCTCACCACCTCTAGGTAGT 3'
fhl09N	5' CTGCTCACTTACCTACTG 3'
fhl10N	5' GGAGCAATATGGTTCGAGAG 3'
fhl12N	5' GAGGTGTGAGGAGGTCTGTC 3'

The deduced gene products of the first eight genes showed similarity to the subunits of molybdopterin oxidoreductases and energy conserving [NiFe] hydrogenases. The protein encoded by the ninth gene (*mnhF*) seemed to be out of our interest. The most important results derived from the BLAST search are summarized in Table 2. The results obtained from the *in silico *analysis are summarized below.

**Table 2:** Properties of the deduced gene products of the *T. litoralis fdh-mhy *operon.

Gene product	molecular mass (kDa)	predicted membrane helices [52]	properties	proposed function	homologues of			% sequence homologies	
					*E.coli *FHL-1	*E. coli *Nuo	*P. abyssi *Hase-4	*T.l*./*E.c*. FHL-1 [Ref.]	*T.l*./*P.a*. [Ref.]
FdhA	71.6	2	molybdopterin cofactor binding motif	formate-dehydrogenase α subunit	Fdh-H		Fdh α	29 [9]	33 [12]
FdhB	18.5	0	2 [4Fe4S] binding site	e^- ^transport protein	HycB		Fdh FeS	33 [9]	55 [12]
MhyC	49	13	NuoL type membrane protein	membrane anchor, proton translocation		NuoL	Hase-4 subunit		53 [12]
MhyD	65.7	15	NuoN type membrane protein	membrane anchor, proton translocation	HycC	NuoN	Comp. B	28 [9]	36 [12]
MhyE	32	5	NuoH type membrane protein	membrane anchor, proton translocation	HycD	NuoH	comp. C	32 [9]	52 [12]
MhyF	62.4	0	conserved CxxC and DPCxxCxxH/R motives	hydrogenase large subunit	HycE	NuoD	comp. G	46 [9]	55 [12]
MhyG	18.7	0	2 [4Fe4S] binding site	e^- ^transport protein	HycF	NuoI	comp. B	36 [9]	51 [12]
MhyH	29.6	1	1 [4Fe4S] binding site	hydrogenase small subunit	HycG	NuoB	comp. I	37 [9]	51 [12]

#### FdhA

The 71.6 kDa protein is similar to the members of the molybdopterin oxidoreductase family, primarily to formate dehydrogenases. It contains one [FeS] cluster binding motif and a molybdopterin cofactor binding site. It is to note, that in many hyperthermophilic archaeal oxidoreductases the molybdenum is replaced by tungsten [24].

#### FdhB

This 18.5 kDa protein contains two ferredoxin-type CxxCxxCxxxC motifs, suggesting the binding of two [4Fe-4S] clusters. It resembles the iron-sulfur binding subunits of different molybdopterin oxidoreductases like the iron-sulfur related protein of *Pyrococcus abyssi *formate dehydrogenase and the HycB subunit of *E. coli *hydrogenase-3. The latter is thought to play role in the electron transport between the formate dehydrogenase and the hydrogenase in the *E. coli *formate hydrogenlyase system (FHL-1) [7,8]. There is also similarity to the electron transport subunit of *Rhodospirillum rubrum *CO dehydrogenase electron transport subunit (CooF), which connects the CO dehydrogenase to the membrane-associated hydrogenase [25].

#### MhyC, D, E

The *mhyCDE *genes code for highly hydrophobic proteins with various numbers of membrane spanning helices (see Table 2). The MhyC (49 kDa), D (65.7 kDa) and E (32 kDa) subunits showed similarity to the NuoL, NuoN, and NuoH membrane proteins of *E. coli*, respectively. All three proteins also resemble the subunits of various Na^+^/H^+ ^antiporters and membrane-bound hydrogenases.

#### MhyF

All conserved motifs characteristic for the large subunits of [NiFe] hydrogenases can be identified in the MhyF, which has 62.4 kDa molecular mass. These include the four cysteines (two CxxC motifs: one at the N- and one at the C-terminal region of the protein) responsible for the binding of the [NiFe] center [26]. At the C-terminal region, the CxxC stretch is part of the DPCxxCxxR motif followed by a C-terminal extension, which is usually cut off by a protease during the posttranslational maturation process [4].

Multiple alignment of the MhyF and the large subunits of various [NiFe] hydrogenases from the group of H_2_-evolving, energy-conserving, membrane-associated hydrogenases revealed an N-terminal extension, which was present only in the MhyF subunit and in the hydrogenase large subunits of the formate hydrogenlyase systems (*E. coli *HycE [7], HyfG [27]). This stretch was proposed to play a role in the interaction between the two enzymes the hydrogenase and the formate dehydrogenase [28].

#### MhyG

The deduced protein of 18.7 kDa is similar to the iron-sulfur cluster containing hydrogenase subunits. The easily recognizable two ferredoxin-type CxxCxxCxxxCP motifs suggested the presence of two [4Fe-4S] clusters, thus MhyG seems to represent an electron transfer subunit. The corresponding subunits of other hydrogenases like HycF in *E. coli*, were proposed to play a role in the electron transport [7,8].

#### MhyH

Based on sequence comparison, the 29.6 kDa MhyH might correspond to the small subunit of [NiFe] hydrogenases. It contains the [4Fe-4S] cluster binding CxxCx_n_GxCxxxGx_m_GCPP (n = 61–101, m = 24–61) motif characteristic for the [NiFe] hydrogenase small subunits [26]. There is no N-terminal leader sequence responsible for TAT-type protein targeting [29], which suggests that the hydrogenase is associated with the cytoplasmic side of the membrane and that the complex has an endogenous substrate.

### *fdhAB-mhyCDEFGH *genes form one transcriptional unit

The genomic organization of the genes suggested that they formed one transcriptional unit. The gene cluster was preceded by a typical archaeal promoter with the TATA box, INR and BRE regions [30]. After the last gene (*mhyH*), an archaeal transcriptional termination signal could be found: a hairpin-loop forming palindrom followed by an oligo(dT) region [31]. To prove that the *fdh*-*mhy *genes form one transcriptional unit, a primer designed to the 3' end of the last gene *mhyH *(fhl8frC1R, see Table 1) was used for cDNA synthesis. PCR was performed with primer pairs that correspond to various regions of the operon (see legend of Fig. 1B and Table 1). The results clearly showed that a single transcript contained all eight genes (Fig. 1B). The presence of alternative transcripts cannot be excluded but there is no indication for other promoters within the operon.

### The expression of *fdh-mhy *genes is up-regulated in cells grown on medium containing peptides

To investigate the physiological role of the Fdh-Mhy proteins, transcriptional regulation of the operon was studied in reverse transcription linked absolute quantification Real-Time PCR experiments.

**Figure 2:**
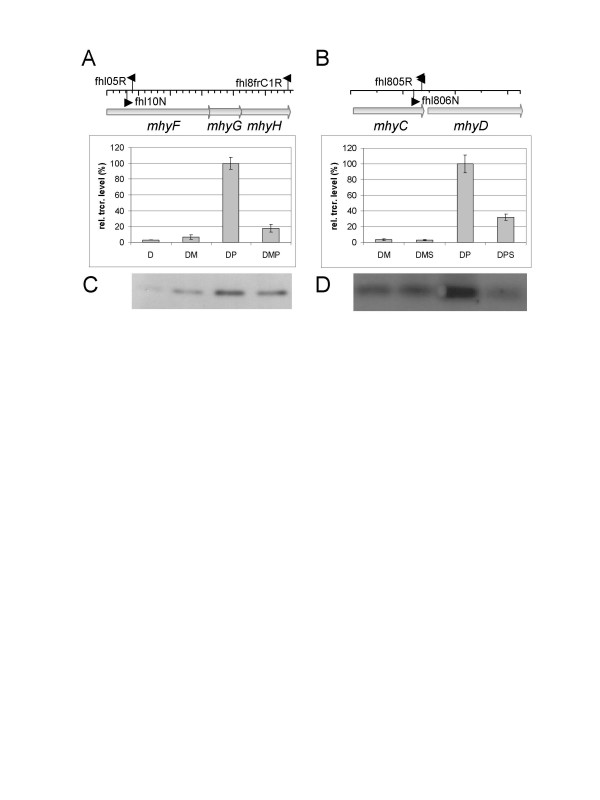
**Gene expression studies with reverse transcription linked absolute quantification Real-Time PCR and immunoblotting**. **A**. Relative transcription level of *fdh-mhy *operon in cells grown on different carbon sources (M and P denotes for the maltose and peptide (casein hydrolysate) content of the medium, respectively, see Materials and Methods). The transcription level of the *fdh-mhy *operon in cells grown on DP medium was taken as 100 %. For reverse transcription RNA was purified from three parallel cultures, cDNA was made using the fhl8frC1R primer designed to the 3' end of *mhyH*. In the Real-Time PCR a primer pair (fhl10N-fhl05R) designed to the *mhyF *gene was used. **B**. Effect of sulfur on the transcription level of *fdh-mhy *operon (M, P and S denotes for the maltose, peptides (casein hydrolysate) and sulfur content of the medium, respectively, see Materials and Methods). For reverse transcription RNA was purified from three parallel cultures, cDNA was made with fhl805R primer designed to the 3' end of *mhyC*. In the Real-Time PCR a primer pair (fhl805R-fhl806N) designed to the *mhyC *was used. **C. D**. Western blotting of the same samples as in A (C) and B (D). In the immunoblotting experiments 20 μg total cellular protein, derived from the same cultures as above, was separated on 12% SDS polyacrylamide gel after boiling in SDS-loading buffer for 10 min. Proteins were blotted on nitrocellulose membrane and the FdhB protein was detected using anti-FdhB antibody.

RNA was isolated from cells grown in the following media: defined medium containing amino acids (D), D supplemented with maltose (DM), peptides (DP) or both (DMP) (see Materials and Methods). The primer used for reverse transcription was in the *mhyH *gene and the quantification was performed by primers located in the *mhyF *gene (see Fig. 2A legend and Table 1). The results are shown in Fig. 2A. The lowest transcription level could be seen in samples grown on defined medium containing amino acids (D) and maltose supplemented D medium (DM). Cells grown on DP medium (containing 0.5% casein hydrolysate) had the highest transcription level, while cells cultivated in DMP medium had a slightly higher gene expression level than in the sample grown on DM. Clearly, if the amount of peptides in the medium is high, the transcription level of the operon is also high (DP); while in the presence of maltose the transcription level is reduced (DMP). Therefore, the physiological function of the complex coded by *fdh-mhy *operon seemed to be related to peptide metabolism.

We also investigated the effect of sulfur on the transcription level of the *fdh-mhy *operon in cells grown on DM (DMS) and DP (DPS) media. Adding sulfur to the medium stimulates the cell growth in certain heterotrophic Archaea. This is probably due to the presence of (an) alternative pathway(s) for removal of the excess reducing power. In these cases, H_2_S is produced beside H_2_. Therefore, the expression level of the hydrogenase genes was expected to depend on sulfur. Primer pairs designed to various parts of the operon (including that pair used in the previous section) gave the same results as the experiments presented. Fig. 2B shows that the presence of sulfur has a negative effect on the expression of the *fdh-mhy *operon in peptide-grown cells, while in the case of maltose containing medium (where the expression is already low) the expression was sulfur independent. The effect was confirmed in both cases by Western blotting using polyclonal antibody raised against FhyB (Fig. 2C, D).

### Cellular localization of the complex

**Figure 3:**
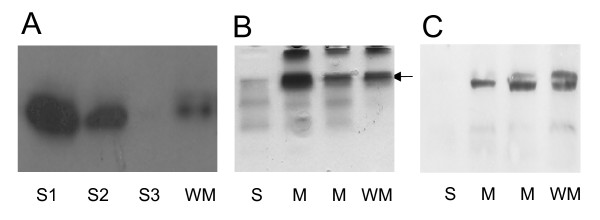
**Cellular localization of FdhB (A), hydrogenase activity staining in native PAGE (B) and immunoblot with anti-FdhB (C)**. **A**. Immunoblotting of *T. litoralis *soluble fraction (S1), supernatant of the first (S2) and second (S3) wash of membrane fraction as well as the washed membrane fraction (WM). Proteins were separated on 12% SDS polyacrylamide gel after boiling in SDS-loading buffer for 10 min and the FdhB subunit was monitored using anti-FdhB antibody. One thousandth of the volume of the prepared fractions was loaded onto the gel to make the signals comparable. **B**. Proteins were separated on 6% native gel and stained for hydrogenase activity. S: soluble fraction M: unwashed membrane fraction WM: washed membrane fraction. The buffer used for the washing step contained 1% dodecyl-β-D-maltoside and 750 mM 6-aminohexanoic acid in 50 mM Bis-Tris pH 7.0. The unsoluble materials were removed by centrifugation at 50000 × g for 20 min and the supernatants were loaded onto the gel. **C**. The proteins from the activity stained gel were transferred to nitrocellulose membrane and screened with anti-FdhB antibody. The hydrogenase activity band corresponding to the FdhB signal is indicated with an arrow.

*In silico *analysis of the Mhy subunits revealed few transmembrane components of the Mhy complex (Table 2). To establish the cellular localization of the Fdh-Mhy complex, soluble and membrane fractions were prepared from *T. litoralis *cells and were investigated by Western blotting technique using anti-FdhB antibody (Fig. 3A). The majority of the FdhB was found in the soluble fraction, but considerable amount of the protein could also be detected in the membrane fractions even after two washing steps (see Materials and Methods). This suggests that FdhB, and likely the other hydrophilic proteins of the complex, are associated – although loosely – to the membrane.

### Hydrogenase activity in *T. litoralis *membrane fraction

The membrane fractions were assayed for hydrogenase activity. The purity of the membrane fractions was checked by measuring the glutamate dehydrogenase activity exclusively present in the cytoplasmic fraction [32]. In each case, less then 2% of the total glutamate dehydrogenase activity was found in the membrane fraction. Strong hydrogenase activity could be detected in the membrane according to both the hydrogen evolution and uptake assays. Furthermore, we could detect formate-dependent H_2 _evolution, but characterization of this activity was difficult due to the extreme oxygen sensitivity of the formate dehydrogenase enzyme.

Sequencing of a part of the so-called membrane-bound hydrogenase operon (*mbh*) indicated the presence of another hydrogenase in the membrane fraction (our unpublished results). To separate the possible different hydrogenases from each other, the cell fractions were run on standard native PAGE [33] and stained for hydrogenase activity. The soluble and the washed membrane fractions showed completely different activity patterns, while in the unwashed membrane fraction, activities specific for both the membrane and the soluble fractions could be seen (Fig. 3B).

Using the anti-FdhB antibody, immunoblot analysis of the activity stained gels was performed to identify the hydrogenase activity band corresponding to the enzyme coded by the *mhy *operon (Fig. 3C). In the immunoblotting experiments, we could detect FdhB, its migration position coincided with one of the membrane fraction specific hydrogenases. FdhB could not be detected in the soluble fraction analyzed in a native gel, although the majority of it could be found in this fraction (see Fig. 3A). We believe that FdhB may have certain physico-chemical properties, which did not allow it to migrate into the native gel. Based on these results, we concluded that comigration of FdhB and the hydrogenase is not accidental but the aftermath of their association.

### Partial purification of the complex

**Figure 4:**
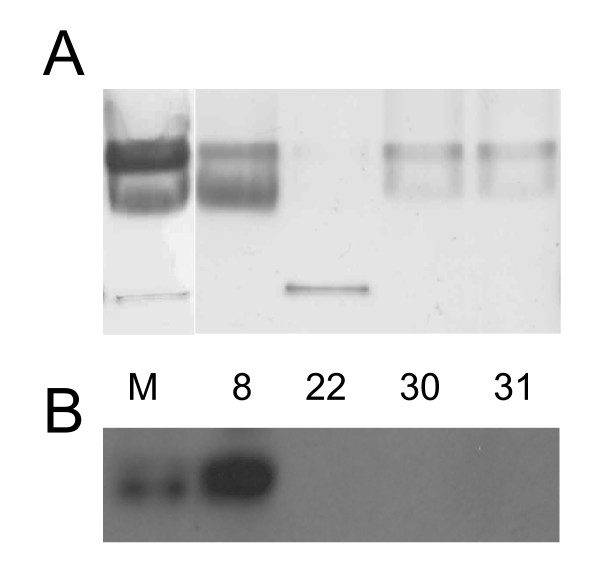
**Activity staining (A) and immunoblot analysis (B) of the CHT chromatography fractions having hydrogenase activity**. **A**. Proteins were separated by 5–13% gradient CN-PAGE and stained for hydrogenase activity. The fractions of the CHT chromatography were concentrated (Millipore Amicon^® ^Ultra centrifugal filter devices, cut-off 100 kDa) then the buffer was replaced by 1% dodecyl-β-D-maltoside and 750 mM 6-aminohexanoic acid in 50 mM Bis-Tris pH 7.0 The unsoluble materials were removed by centrifugation at 50000 × g for 20 min and the supernatants were loaded onto the gel. **B**. Proteins were separated on 12% SDS polyacrylamide gel after boiling in SDS-loading buffer for 10 min, transferred to nitrocellulose membrane and screened with anti-FdhB antibody. M fr.: membrane fraction that was loaded onto the CHT column. The numbers mean the numbers of the fractions analyzed.

Unfortunately, the purification of the complex to homogenity failed even when purification was attempted in an anaerobic workstation. The hydrogenase enzyme quickly lost its activity upon exposure to oxygen and only one chromatography step could be performed without losing the activity. Various chromatographic methods were attempted and the best results were obtained with ceramic hydroxyapatite chromatography (see Materials and Methods). Fractions eluted from the ceramic hydroxyapatite column were assayed for hydrogen evolution activity. The active fractions (fr.8., fr.22, fr.30–31) were analyzed both by native CN-PAGE [34] with activity staining (Fig. 4A) and by Western blotting (Fig. 4B). The results clearly showed that the FdhB protein and the membrane-bound hydrogenase copurified: both the hydrogenase activity and FdhB immuno-signal could be detected in the fraction 8. The hydrogenase activity in the fraction 22 could be another membrane-bound hydrogenase or a FdhB free form of the Fdh-Mhy complex. It seems that a small portion of the hydrogenase part of the Mhy complex is bound to the column and eluted in fraction 30 and 31. The reason for this phenomenon is unclear.

## Discussion

The presence of multiple hydrogenases in microorganisms implies that various hydrogenases may be linked to alternative metabolic pathways. So far, only one hydrogenase, the cytoplasmic hydrogenase-1 (Hyh1), was characterized from *T. litoralis *[22]. However, the genes of Hyh2 (GenBank accession no.: EU024408) and a region of the membrane-bound hydrogenase (*mbh*) genes were also identified (our unpublished results).

An operon, consisting of eight genes (*fdhAB-mhyCDEFGH*), was isolated and sequenced from *T. litoralis*. The operon codes for a formate dehydrogenase and a [NiFe] hydrogenase which belongs to the group of H_2_-evolving, energy-conserving membrane-associated hydrogenases. Remarkably, the operon could be found only in *P. abyssi *among the four closely related species (*P. furious *[35], *P. abyssi *[36], * Pyrococcus horikoshii *[37], * Thermococcus kodakaraensis *[38]) whose genomes were sequenced, although the other soluble and membrane-associated hydrogenases are widespread among the members of the Thermococcaceae family.

The genomic context and transcriptional analysis of the genes indicated that the Fdh-Mhy proteins form a functional complex and *in silico *analysis predicted that few subunits (MhyCDE) are integrated membrane proteins. Western blotting experiments using anti-FdhB antibody revealed that the hydrophilic FdhB protein is also attached to the membrane, although large portion of FdhB could be detected in the soluble fraction. Protein purification and separation experiments, combined with activity staining and immunological detection, were performed to demonstrate that the membrane-bound hydrogenase was associated with the formate dehydrogenase subunits. Formate driven hydrogen evolution could also be detected, but this activity was not fully reproducible, probably due to protein sensitivity. Comprehensive *in silico *analysis made it unlikely, that except for formate, any other substrate of the FdhAB dimer (nitrate, DMSO or TMA) could be considered. Therefore, it was concluded, that the FdhAB-MhyCDEFGH proteins formed a membrane-bound formate dehydrogenase coupled hydrogenase (FDH-MHY) complex, although the subunits of the formate dehydrogenase seemed to be dissociable from the other part of the complex.

There are a few reactions or pathways leading to formate formation in various microbes including the pyruvate [39], the methane [40], the glyoxylate and dicarboxylate [41,42] and the amino acid metabolism [43]. Formate should be present also in the metabolism in these cells, as usually at least one formate dehydrogenases can be found in the members of the Thermococcaceae family [35-38].

In *E. coli *the formate hydrogenlyase is responsible for the removal of formate to prevent the cytoplasm from acidification [39]. Formate is generated from pyruvate by the pyruvate formate lyase enzyme [44]. Searching for the four known hyperthermophilic genomes, we could find pyruvate formate lyase (PFL) only in *T. kodakaraensis*, but not in *P. abyssi*, where the *fdh-mhy *homologous genes are present. Instead, in the known members of Thermococcaceae family usually pyruvate is oxidized by a pyruvate-ferredoxin oxidoreductase (PFOR) [45] leading to the formation of reduced ferredoxin, which is utilized directly by the membrane-bound hydrogenase (Mbh) [21]. Alternatively, the reduced ferredoxin can be converted to NAD(P)H by ferredoxin:NAD(P) oxidoreductase (FNOR) and the reduced NAD(P)H serves as substrate for the cytoplasmic heterotetrameric hydrogenases [21,46]. Therefore, it seems that the pyruvate metabolism is strongly linked to the hydrogen metabolism (Mbh and soluble hydrogenases) via ferredoxin produced by the PFOR, but no indication could be found for production of formate from pyruvate. Presuming that FDH-MHY are linked to similar pathways for both *P. abyssi *and *T. litoralis*, it seems unlikely that pyruvate is the formate donor for the FDH-MHY complex in these microbes.

Moreover, gene expression study disclosed that the complex is highly upregulated (more than one order of magnitude) in cells grown on peptide containing medium (DP medium) as compared to the samples grown on medium containing only amino acids (D) or D supplemented with maltose (DM). Hyperthermophilic heterotrophic microorganisms usually show poor growth on medium containing single amino acids. This might be due to either the restricted capacity of the cells to take up several essential amino acids or the greater thermal instability of single amino acids as compared to the peptides, or both [47]. This might explain the low expression level in D medium. Most hyperthermophilic heterotrophs, including *T. litoralis*, are known to prefer peptide over carbohydrates, but addition of maltose to the peptide containing media was reported to stimulate growth [9]. Consequently, in these cases both type of carbon sources are utilized. This might elucidate the reduced level of the *fdh-mhy *mRNA in carbohydrate supplemented peptide containing media (DMP). In the case of DM medium, the cells use maltose as main carbon source instead of amino acids and under these conditions the *fdh-mhy *genes were weakly transcribed. It is to note that the *fdh-mhy *transcript level in the cells grown in DM medium is slightly higher than in the cultures cultivated in basic (D) medium. However, this increase is negligible as compared to the activation occured in the samples grown in the presence of peptides (DP). No obvious explanation can be given for this slight – but detectable – activation by maltose. Therefore, it was concluded that the FHL complex is linked to the peptide rather than to the carbohydrate metabolism. Addition of sulfur to the medium suppressed the induction by peptides, probably due to the appearance of alternative, more favorized pathways.

Unfortunately, the amino acid metabolism is not well understood in hyperthermophilic archaea. Transaminases and four distinct 2-keto acid oxidoreductases are involved in the conversion of amino acids into their corresponding coenzyme A derivatives [12]. There are pathways, in which 2-keto acids generated from amino acids are decarboxylated to aldehydes and then further oxidized to carboxylic acids [47]. Two aldehyde oxidizing enzymes were isolated from *T. litoralis*, these are the aldehyde:ferredoxin oxidoreductase (AOR) and the formaldehyde:ferredoxin oxidoreductase (FOR) [15,24]. FOR can convert only C1-C3 aldehydes *in vitro*, its physiological function is not completely understood, but believed to participate in the catabolism of basic amino acids [48]. Moreover, *in vitro *both AOR and FOR can use formaldehyde as a substrate and produce formate [15,24], therefore enzymes for the endogenous formation of formate are potentially present in *T. litoralis*.

## Conclusion

In *T. litoralis*, the presence of a formate dehydrogenase associated [NiFe] hydrogenase (formate hydrogenlyase) complex was demonstrated which was likely involved in the removal of formate generated during peptide catabolism. This might have an important practical consequence. In a two stage system converting keratin-containing biowaste to biohydrogen, *T. litoralis *showed better performance than *P. furiosus *[23] and this "amino acid – formate – hydrogen" pathway might be responsible for this better efficacy.

## Methods

### Strains and growth conditions

*Thermococcus litoralis *DSM 5473 was maintained in complex medium (CM) under strictly anaerobic conditions at 85°C [22]. For the gene expression experiments, cells were grown in defined medium (D), in D supplemented with 0.5 % (wt/vol) maltose (DM) or 0.5 % (wt/vol) casein hydrolysate (DP) or maltose and casein hydrolysate (DMP). If sulfur was also added in a 1 % concentration, it is indicated by "S".

D medium (per liter): NaCl 24 g, MgCl_2 _× 6H_2_O 10.6 g, Na_2_SO_4 _4 g, CaCl_2 _× 2H_2_O 1.5 g, KCl 0.7 g, NaHCO_3_0.2 g, KBr 0.1 g, SrCl_2 _25 mg, H_3_BO_3 _30 mg, Na_2_WO_4 _× 2H_2_O 3 mg, K_2_HPO_4 _140 mg, FeSO_4 _× 7H_2_O 1.4 mg, MnSO_4 _× 7 H_2_O 0.5 mg, CoSO_4 _× 7 H_2_O 0.36 mg, NiCl_2 _× 6 H_2_O 0.2 mg, Na_2_MoO_4 _× 2 H_2_O 1 μg, CuSO_4 _× 5 H_2_O 1 μg, pyridoxine-HCl 0.1 mg, *p*-aminobenzoic acid 0.05 mg, nicotinic acid 0.05 mg, DL-Ca-panthotenate 0.05 mg, thiamine-HCl 0.05 mg, DL-6,8-lipoic acid 0.05 mg, riboflavin 0.04 mg, biotin 0.2 mg, folic acid 0.02 mg, 200 mg of each of the 20 amino acids, adenine 10 mg, uracil 10 mg, resazurin 0.2 mg, cysteine 0.4 g, (pH 6.5).

*E. coli *XL1-Blue MRF' and *E. coli *BL21(DE3) CodonPlus-RIL (Stratagene) cells were used for cloning and protein overexpression, respectively. *E. coli *strains were cultivated in Luria-Bertani medium [33] at 37°C, antibiotics were used at the following concentrations (μg·ml^-1^): ampicillin (100), kanamycin (50), and tetracycline (10).

### DNA manipulation techniques

All cloning and DNA manipulation steps were performed following the standard practice [49]. The isolated DNA fragments were usually subcloned into pBluescript SK+/- (Stratagene) vector. Southern blotting, hybridization, colony and plaque hybridizations were performed according to the standard practice [33] and the manufacturer's instructions (Roche). The probes were digoxigenin labeled and visualized colorimetrically as described by the supplier (Roche).

### Isolation of the *fdh-mhy *operon and sequence analyses

Two genes encoding for the formate dehydrogenase subunits were sequenced previously [22]. For sequencing the whole cluster, *T. litoralis *partial genomic DNA library from *Eco*RI-*Xba*I digested genomic DNA was prepared in pBluescript SK+ vector. The labeled insert of pLHU1/1 clone was used to fish out the clone pFhl6 (Fig. 1A.), which was further subcloned and sequenced. As a next step, the labeled fragment of the pFhl10 was used to isolate the pFhl17 clone from *Bam*HI-*Eco*RV *T. litoralis *partial genomic DNA library. These fragments were subcloned and  their nucleotide sequences were  determined on both strands by an Applied Biosystems 373 Stretch DNA sequencer.

### Bioinformatics tools

Comparison of nucleotide and amino acid sequences were carried out with BLAST programs [50]. Multiple alignments were done with ClustalW 1.8 [51]. Membrane spanning helices were predicted by the HMMTOP software [52,53].

### RNA isolation

Cells grown to mid-logarithmic phase were harvested and total RNA was extracted using Tri-Reagent™ (Sigma) following the manufacturer's instructions. RNA was further treated with RNase-free DNaseI to remove any residual DNA and was quantified by NanoDrop ND-1000 spectrophotometer (NanoDrop Technologies). The integrity of the RNA preparation was verified by gel electrophoresis according to Ausubel *et al*. [33].

### Reverse transcription, RT-PCR

From the DNA-free RNA preparation typically 1 μg RNA was reverse transcribed into cDNA using RevertAid™ H Minus M-MuLV Reverse Transcriptase (Fermentas) (in 20 μl final volume) and 1 μl aliquot of this mixture was used in the amplification reactions. Negative control reactions were performed by omitting the reverse transcriptase. The primers used for the reverse transcription and amplification reactions are listed in Table 1. PCR was done in PCRExpress thermocycler (Hybaid).

### Real-Time PCR

Absolute quantification Real-Time PCR was performed in 7500 Real-Time PCR System (Applied Biosystems), using SYBR^® ^Green PCR Master Mix (Applied Biosystems). In the absolute quantification experiments a tenfold dilution series of the plasmids (pFhl7 and pFhl17) harboring the genomic region of interest was used for calibration.

### Anti-FdhB antibody preparation

His-tagged FdhB was overexpressed in *E. coli *BL21(DE3) CodonPlus-RIL (Stratagene) using pET32a vector (Novagene), and purified on IMAC column as described previously [33]. Polyclonal antibody was produced in rabbits. The immunization and the recovery of the IgG fraction from the serum were carried out according to standard methods [33]. The crude IgG fraction was further purified on DEAE Affi-Gel Blue Gel column (Bio-Rad). Preimmune serum was also prepared and used in control experiments to prove the specificity of the Anti-FdhB antibody. Both ELISA and Western-blotting showed that the Anti-FdhB antibody specifically interacted with FdhB.

### Protein separation, Western blotting and hybridization

SDS-PAGE and native PAGE were executed according to Ausubel *et al*. [33], Colourless Native PAGE (CN-PAGE) was performed as described by Schagger *et al*. [34]. Proteins were transferred to ECL+ Nitrocellulose membrane (Amersham Biosciences) from polyacrylamide gel using Trans-Blot^® ^SD Semi-Dry Electrophoretic Transfer Cell (BioRad). Membranes were hybridized with anti-FdhB antibody (1:100 diluted, protein concentration: 6 mg/ml) and incubated with 1:10000 diluted horseradish peroxidase conjugated anti-rabbit secondary antibody (Amersham Biosciences). Reporter protein activity was detected using ECL plus Western Blotting Detection System (Amersham Biosciences).

### Preparation of cell extracts

*T. litoralis *cells suspended in 50 mM Tris-HCl buffer (pH 8.0) were disrupted with a French Pressure Cell (ThermoIEC, French^® ^Press) at 20000 psi. The cell debris was removed with centrifugation at 20000 × g for 20 min. The supernatant was centrifuged at 100000 × g, at 4°C for 1.5 hours. The pellet was washed twice with the same buffer as above and was considered as membrane fraction.

### Enzyme assays

Hydrogen uptake activity was measured anaerobically by H_2_-dependent reduction of benzyl viologen (0.4 mM) in 50 mM Tris-HCl buffer (pH 8.0) in rubber stopper-sealed glass cuvettes at 80°C. The absorbance changes were recorded in Unicam UV/VIS-UV2 spectrophotometer at 600 nm. H_2 _evolution was measured using methyl viologen (3 mM) as electron donor in 50 mM Tris-HCl buffer (pH 8.0) at 80°C by gas chromatography (model 6890N, Agilent Technologies). The reaction was started by adding sodium dithionite (up to 30 mM final concentration).

Hydrogenase activity staining of native polyacrylamide gels: the gel was placed in a flask containing 200 ml of 50 mM Tris-HCl (pH 8.0) buffer and 1 mM benzyl viologen. It was flushed with nitrogen for 20 min then with hydrogen at 85°C. After the appearance of the activity bands 2,3,5-triphenyl-tetrazolium-chlorid was added to a final concentration of 20 mM to stabilize the staining.

Glutamate dehydrogenase activity was followed by glutamate driven reduction of NADP^+ ^measured at 340 nm in 20 mM sodium phosphate buffer (pH 7.0) containing 6 mM sodium glutamate and 0.4 mM NADP^+ ^[32].

### Ceramic hydroxyapatite chromatography

Membrane fractions (prepared in 20 mM phosphate buffer (pH 7.0) instead of Tris-HCl as above) were loaded onto a ceramic hydroxyapatite column (CHT^® ^Ceramic Hydroxyapatite (BioRad)) equilibrated with 10 mM potassium phosphate buffer (pH 7.0) containing 2 mM DTT (in few cases 1% dodecyl-β-D-maltoside and 750 mM 6-aminohexanoic acid in 50 mM Bis-Tris pH 7.0. were used for solubilization, but they did not improve the separation). Proteins were eluted using a linear gradient from 10 to 500 mM potassium phosphate. Anaerobic protein purification was done in a Bactron IV (Shel-Lab) anaerobic chamber.

### Nucleotide sequence accession number

The 10474 bp long sequence containing *fdhAB-mhyCDEFGH *genes was deposited in GenBank under the accession number AF039208.

## Abbreviations

AOR: aldehyde:ferredoxin oxidoreductase, BRE: transcription factor B recognition element, CHT: Ceramic Hydroxyapatite, Coo: CO-oxidizing:H_2_-forming enzyme system in *Rhodospirillum rubrum *or *Carboxydothermus hydrogenoformans*, Fdh: formate dehydrogenase, FHL: formate hydrogenlyase, FOR: formaldehyde:ferredoxin oxidoreductase, Hyc: energy-converting [NiFe] hydrogenase (part of FHL-1) in *Escherichia coli*, Hyf: energy-converting [NiFe] hydrogenase (part of FHL-2) in *Escherichia coli*, Hyh: cytoplasmic NADP-reducing [NiFe] hydrogenase in *Thermococcus litoralis *and *Pyrococus furiosus*, FNOR: ferredoxin:NAD(P) oxidoreductase, INR: initiator element, Mbh: membrane-bound [NiFe] hydrogenase of *Pyrococcus furiosus *type, Mhy: membrane-bound [NiFe] hydrogenase linked to Fdh, Nuo: NADH:ubiquinone oxidoreductase, MNH: multisubunit Na+/H+ antiporter, PFOR: pyruvate-ferredoxin oxidoreductase, TAT: twin-arginine translocation pathway.

## Authors' contributions

MT carried out the isolation of *fdh-mhy *operon, did the Western blot and Real-Time experiments and measured the enzyme activities. AT did the RT-PCR and helped to design the Real-Time PCR experiments. BB overexpressed and purified the FdhB protein for antibody production. Chromatography steps during protein purification were done by AV. GR took part in the isolation of *fdh-mhy *operon and participated in the design of the study and helped to draft the manuscript. KLK participated in the design and coordination of the study and helped to draft the manuscript.

## References

[B1] Stetter KO (1996). Hyperthermophilic prokaryotes. FEMS Microbiol Rev.

[B2] Woese CR, Kandler O, Wheelis ML (1990). Towards a natural system of organisms: proposal for the domains of Archaea, Bacteria and Eucarya. Proc Natl Acad Sci USA.

[B3] Ma K, Schicho RN, Kelly RM, Adams MWW (1993). Hydrogenase of the hyperthermophile *Pyrococcus furiosus *is an elemental sulfur reductase or sulfhydrogenase: evidence for a sulfur-reducing hydrogenase ancestor. Proc Natl Acad Sci USA.

[B4] Vignais PM, Colbeau A (2004). Molecular biology of microbiol hydrogenases. Curr Issues Mol Biol.

[B5] Rákhely G, Kovács ÁT, Maróti G, Fodor BD, Csanádi Gy, Latinovics D, Kovács KL (2004). Cyanobacterial-type, heteropentameric, NAD+-reducing NiFe hydrogenase in the purple sulfur photosynthetic bacterium *Thiocapsa roseopersicina*. Appl Environ Microbiol.

[B6] Hedderich R (2004). Energy-converting [NiFe] hydrogenases from archaea and extremophiles: ancestors of complex I. J Bioenerg Biomemb.

[B7] Böhm R, Sauter M, Böck A (1990). Nucleotide sequence and expression of an operon in *Escherichia coli *coding for formate hydrogenlyase components. Mol Microbiol.

[B8] Sauter M, Böhm R, Böck A (1992). Mutational analysis of the operon (*hyc*) determining hydrogenase 3 formation in *Escherichia coli*. Mol Microbiol.

[B9] Neuner A, Jannasch HW, Belkin S, Stetter KO (1990). *Thermococcus litoralis *sp. nov.: A new species of extremely thermophilic marine archaebacteria. Arch Microbiol.

[B10] Huber R, Stetter KO (2001). Discovery of hyperthermophilic microorganisms. Methods Enzymol.

[B11] Kengen SWM, Stams AJM, de Vos WM (1996). Sugar metabolism of hyperthermophiles. FEMS Microbiol Rev.

[B12] Mai X, Adams MWW (1996). Characterization of a forth type of 2-keto acid-oxidizing enzyme from a hyperthermophilic archaeon: 2-ketoglutarate ferredoxin oxidoreductase from *Thermococcus litoralis*. J Bacteriol.

[B13] Mai X, Adams MWW (1996). Purification and characterization of two reversible and ADP-dependent acetyl coenzyme A synthetases from the hyperthermophilic archaeon *Pyrococcus furiosus*. J Bacteriol.

[B14] Mukund S, Adams MWW (1991). The novel tungsten-iron-sulfur protein of the hyperthermophilic archaebacterium *Pyrococcus furiosus*, is an aldehyde ferredoxin oxidoreductase. J Biol Chem.

[B15] Mukund S, Adams MWW (1993). Characterization of a novel tungsten-containing formaldehyde ferredoxin oxidoreductase from the hyperthermophilic archaeon, *Thermococcus litoralis*. J Biol Chem.

[B16] Roy R, Mukund S, Schut GJ, Dunn DM, Weiss R, Adams MWW (1999). Purification and molecular characterization of the tungsten-containing formaldehyde ferredoxin oxidoreductase from the hyperthermophilic archaeon *Pyrococcus furiosus*: the third of a putative five-member tungstoenzyme family. J Bacteriol.

[B17] Bryant FO, Adams MWW (1989). Characterization of hydrogenases from the hyperthermophilic Archaebacterium, *Pyrococcus furiosus*. J Biol Chem.

[B18] Ma K, Weiss R, Adams MWW (2000). Characterization of hydrogenase II from the hyperthermophilic archaeon *Pyrococcus furiosus *and assessment of its role in sulfur reduction. J Bacteriol.

[B19] Sapra R, Verhagen MF, Adams MWW (2000). Purification and characterization of a membrane-bound hydrogenase from the hyperthermophilic archaeon *Pyrococcus furiosus*. J Bacteriol.

[B20] Sapra R, Bagramyan K, Adams MWW (2003). A simple energy-conserving system: proton reduction coupled to proton translocation. Proc Natl Acad Sci USA.

[B21] Silva PJ, Ban ECD van den, Wassink H, Haaker H, de Castro B, Robb FT, Hagen WR (2000). Enzymes of hydrogen metabolism in *Pyrococcus furiosus*. Eur J Biochem.

[B22] Rákhely G, Zhou ZH, Adams MWW, Kovács KL (1999). Biochemical and molecular characterization of the [NiFe] hydrogenase from the hyperthermophilic archaeon, *Thermococcus litoralis*. Eur J Biochem.

[B23] Bálint B, Bagi Z, Tóth A, Rákhely G, Perei K, Kovács K (2005). Utilization of keratin-containing biowaste to produce biohydrogen. Appl Microbiol Biotechnol.

[B24] Kletzin A, Adams MWW (1996). Tungsten in biological systems. FEMS Microbiol Rev.

[B25] Ensign SA, Ludden PW (1991). Characterization of the CO oxidation/H_2 _evolution system of *Rhodospirillum rubrum*: role of a 22-kDa iron-sulfur protein in mediating electron transfer between carbon monoxide dehydrogenase and hydrogenase. J Biol Chem.

[B26] Albracht SPJ (1994). Nickel hydrogenases: in search of the active site. Biochim Biophys Acta.

[B27] Andrews SC, Berks BC, Mcclay J, Ambler A, Quail MA, Golby P, Guest JR (1997). A 12- cistron *Escherichia coli *operon (*hyf*) encoding a putative proton- translocating formate hydrogenlyase system. Microbiology.

[B28] Künkel A, Vorholt JA, Thauer RK, Hedderich R (1998). An *Escherichia coli *hydrogenase-3-type hydrogenase in methanogenic archaea. Eur J Biochem.

[B29] Lee PA, Tullman-Ercek D, Georgiou G (2006). The bacterial twin-arginine translocation pathway. Annu Rev Microbiol.

[B30] Soppa J (1999). Transcription initiation in Archaea: facts, factors and future aspects. Mol Microbiol.

[B31] Brown JW, Daniels CJ, Reeve JN (1989). Gene structure, organization, and expression in archaebacteria. Crit Rev Microbiol.

[B32] Ma K, Robb FT, Adams MWW (1994). Purification and characterization of NADP-specific alcohol dehydrogenase and glutamate dehydrogenase from the hyperthermophilic archaeon *Thermococcus litoralis*. Appl Env Microbiol.

[B33] Ausubel FM, Brent R, Kingston RE, Moore DD, Seidman JG, Smith JA, Struhl K (1996). Current Protocols in Molecular Biology.

[B34] Schagger H, Cramer WA, Jagow G (1994). Analysis of molecular masses and oligomeric states of protein complexes by blue native electrophoresis and isolation of membrane protein complexes by two-dimensional native electrophoresis. Anal Biochem.

[B35] Maeder DL, Weiss RB, Dunn DM, Cherry JL, Gonzalez JM, DiRuggiero J, Robb FT (1999). Divergence of the hyperthermophilic archaea *Pyrococcus furiosus *and *P. horikoshii *inferred from complete genomic sequences. Genetics.

[B36] Cohen GN, Barbe V, Flament D, Galperin M, Heilig R, Lecompte O, Poch O, Prieur D, Querellou J, Ripp R, Thierry J-C, Oost J Van der, Weissenbach J, Zivanovic Y, Forterre P (2003). An integrated analysis of the genome of the hyperthermophilic archaeon *Pyrococcus abyssi*. Mol Microbiol.

[B37] Kawarabayasi Y, Sawada M, Horikawa H, Haikawa Y, Hino Y, Yamamoto S, Sekine M, Baba S, Kosugi H, Hosoyama A, Nagai Y, Sakai M, Ogura K, Otuka R, Nakazawa H, Takamiya M, Ohfuku Y, Funahashi T, Tanaka T, Kudoh Y, Yamazaki J, Kushida N, Oguchi A, Aoki K, Nakamura Y, Robb TF, Horikoshi K, Masuchi Y, Shizuya H, Kikuchi H (1998). Complete sequence and gene organization of the genome of a hyper-thermophilic archaebacterium, *Pyrococcus horikoshii *OT3. DNA Res.

[B38] Fukui T, Atomi H, Kanai T, Matsumi R, Fujiwara S, Imanaka T (2005). Complete genome sequence of the hyperthermophilic archaeon *Thermococcus kodakaraensis *KOD1 and comparison with *Pyrococcus *genomes. Genome Res.

[B39] Böck A, Sawers G, Neidhart FC (1996). Fermentation. Escherichia coli and Salmonella typhimurium: cellular and molecular biology.

[B40] Bodrossy L, Kovács KL (1994). Methane utilizing bacteria and their biotechnological applications. Indian J Exp Biol.

[B41] Dimroth P, Schink B (1998). Energy conversion in the decarboxylation of dicarboxylic acids by fermenting bacteria. Arch Microbiol.

[B42] Kornberg HL (1966). The role and control of the glyoxylate cycle in *Escherichia coli*. Biochem J.

[B43] Hesslinger C, Fairhurst SA, Sawers G (1998). Novel keto acid formate-lyase and propionate kinase enzymes are components of an anaerobic pathway in *Escherichia coli *that degrades L-threonine to propionate. Mol Microbiol.

[B44] Knappe J, Sawers G (1990). A radical-chemical route to acetyl-CoA: the anaerobically induced pyruvate formate-lyase system of *Escherichia coli*. FEMS Microbiol Rev.

[B45] Blamey JM, Adams MWW (1993). Purification and characterization of pyruvate ferredoxin oxidoreductase from the hyperthermophilic archaeon, *Pyrococcus furiosus*. Biochim Biophys Acta.

[B46] Ma K, Zhou ZH, Adams MWW (1994). Hydrogen production from pyruvate by enzymes purified from the hyperthermophilic archaeon, *Pyrococcus furiosus: *A key role for NADPH. FEMS Microbiol Lett.

[B47] Rinker KD, Kelly RM (1996). Growth physiology of the hyperthermophilic *Thermococcus litoralis*: development of a sulfur-free defined medium, characterization of an exopolysaccharide, and evidence of biofilm formation. Appl Env Microbiol.

[B48] Adams MWW, Holden JF, Menon AL, Schut GJ, Grunden AM, Hou C, Hutchins AM, Jenney FE, Kim C, Ma K, Pan G, Roy R, Sapra R, Story SV, Verhagen MFJM (2001). Key rule for sulfur in peptide metabolism and in regulation of three hydrogenases in the hyperthermophilic archaeon Pyrococcus furiosus. J Bacteriol.

[B49] Sambrook J, Fritsch EF, Maniatis T (1989). Molecular cloning: a Laboratory Manual.

[B50] The NCBI HomePage. http://www.ncbi.nlm.nih.gov.

[B51] The BCM Search Launcher: Multiple Sequence Alignments. http://searchlauncher.bcm.tmc.edu/multi-align/multi-align.html.

[B52] HMMTOP. http://www.enzim.hu/hmmtop/index.html.

[B53] Tusnády GE, Simon I (2001). The HMMTOP transmembrane topology prediction server. Bioinformatics.

